# Implementation of robotic rectal surgery training programme: importance of standardisation and structured training

**DOI:** 10.1007/s00423-018-1690-1

**Published:** 2018-06-20

**Authors:** Sofoklis Panteleimonitis, Sotirios Popeskou, Mohamed Aradaib, Mick Harper, Jamil Ahmed, Mukhtar Ahmad, Tahseen Qureshi, Nuno Figueiredo, Amjad Parvaiz

**Affiliations:** 1 Poole Hospital NHS Trust, Longfleet Road, Poole, BH15 2JB UK; 2 School of Health Sciences and Social Work, University of Portsmouth, James Watson West, 2 King Richard 1st Road, Portsmouth, PO1 2FR UK; 3 Bournemouth University School of Health and Social Care, Bournemouth, UK; 4 Champalimaud Foundation, Av. Brasilia, 1400-038 Lisbon, Portugal

**Keywords:** Robotic surgery, Rectal surgery, da Vinci xi, Training, Standardisation

## Abstract

**Purpose:**

A structured training programme is essential for the safe adoption of robotic rectal cancer surgery. The aim of this study is to describe the training pathway and short-term surgical outcomes of three surgeons in two centres (UK and Portugal) undertaking single-docking robotic rectal surgery with the da Vinci Xi and integrated table motion (ITM).

**Methods:**

Prospectively, collected data for consecutive patients who underwent robotic rectal cancer resections with the da Vinci Xi and ITM between November 2015 and September 2017 was analysed. The short-term surgical outcomes of the first ten cases of each surgeon (supervised) were compared with the subsequent cases (independent). In addition, the Global Assessment Score (GAS) forms from the supervised cases were analysed and the GAS cumulative sum (CUSUM) charts constructed to investigate the training pathway of the participating surgeons.

**Results:**

Data from 82 patients was analysed. There were no conversions to open, no anastomotic leaks and no 30-day mortality. Mean operation time was 288 min (SD 63), median estimated blood loss 20 (IQR 20–20) ml and median length of stay 5 (IQR 4–8) days. Thirty-day readmission and reoperation rates were 4% (*n* = 3) and 6% (*n* = 5) respectively. When comparing the supervised cases with the subsequent solo cases, there were no statistically significant changes in any of the short-term outcomes with the exception of mean operative time, which was significantly shorter in the independent cases (311 vs 275 min, *p* = 0.038). GAS form analysis and GAS CUSUM charting revealed that ten proctoring cases were enough for trainee surgeons to independently perform robotic rectal resections with the da Vinci Xi.

**Conclusions:**

Our results show that by applying a structured training pathway and standardising the surgical technique, the single-docking procedure with the da Vinci Xi is a valid, reproducible technique that offers good short-term outcomes in our study population.

## Introduction

Robotic rectal surgery has increasingly gained acceptance since the first robotic colectomy in 2002 [1]. Its increasing adoption is evident from the growing number of studies published on the subject [2–4]. Although 3D views and angulated instruments with multiple degrees of freedom might confer an advantage for patients having robotic rectal surgery, appropriate training and a standardised approach remain imperative for improved clinical outcomes. Currently, various approaches are practiced, such as the hybrid, reverse hybrid and single- or double-docking techniques [5–12]. The favoured method is the single-docking totally robotic approach, as it eliminates the need for repeated docking or undocking of the robot and at the same time preserves the advantages of utilising the robot for the entire procedure [5, 7, 13]. This approach was feasible with the da Vinci Si® (*Intuitive Surgical, Sunnyvale, USA*) system with a change in port configuration between the stages of abdominal and pelvic dissection, as described in previous publications [7, 14].

The da Vinci Xi® developed by *Intuitive Surgical* is the fourth-generation robotic system affording several improvements. Advancements in technology combined with the ability to support new integrated technologies such as the integrated table motion (ITM) enable the surgeon to perform single-docking robotic surgery without having to change the port configuration. In addition, a new laser target system, reduced camera size to 8 mm, redesigned patient cart with new overhead instrument arm architecture and thinner, longer arms serve to improve the operative experience of the surgeon and the perioperative team. The ITM system is a unique feature which enables table movement while the patient cart is docked enabling easier mobilisation of the splenic flexure due to the head end of the table being raised during dissection [15, 16]. This also reduces the time in which the patient remains in the steep Trendelenburg position, reducing the risk of adverse perioperative effects such as deep vein thrombosis, ophthalmologic complications and complications secondary to increased intracranial pressure and laryngeal oedema [17–19].

We describe our experience of the safe implementation and adoption of robotic rectal surgery with the da Vinci Xi® and ITM at two centres applying a structured training model and standardised approach to rectal surgery. This study describes the training pathway and short-term clinical outcomes of the first 82 patients treated using this approach. Furthermore, the supervised cases (*n* = 30) performed by the three surgeons are compared with the independently performed rectal resections (*n* = 52) performed after completion of their training. Finally, we examine the Global Assessment Score (GAS) forms of the supervised cases and investigate the GAS cumulative sum (CUSUM) graphs for each surgeon. We aim to exhibit the feasibility and safety of this training pathway and demonstrate the adoption of totally robotic single-docking rectal surgery with the da Vinci Xi and ITM.

## Methods

Data was collected prospectively and stored in a secure, designated database since the start of the training programme in November 2015. During the study period from November 2015 to September 2017, 82 consecutive patients with a known diagnosis of adenocarcinoma of the rectum were operated on in two centres, one in the UK and one in Portugal. A standardised approach of single-docking totally robotic rectal resection surgery with the da Vinci Xi® and ITM was used [20]. Demographic, clinical and pathological data on all patients operated on were collected prospectively and stored in a secure database. In addition, we recorded the time the patients spent in the reverse Trendelenburg position during splenic flexure mobilisation for the first 20 cases.

All procedures were performed by three surgeons under the direct supervision of a single trainer (surgeon ACP). The trainer (ACP) had performed more than 500 laparoscopic rectal resections before undertaking robotic colorectal surgery, and his robotic colorectal experience included performing or teaching over 300 colorectal resections. Prior to performing surgery with the da Vinci Xi and ITM, formal training for the new platform was undertaken. Transitioning to the new robotic platform was straightforward considering the trainer’s experience with the previous system and due to the standardisation of operative technique. All three trainee surgeons had previously undertaken formal training in minimally invasive colorectal surgery, with all surgeons having performed more than 30 laparoscopic rectal resections each. None of the surgeons had any prior robotic surgery experience. The first ten cases of each surgeon were performed under the direct supervision of the single trainer, while successive cases were performed independently by each surgeon. The baseline characteristics and short-term surgical outcomes of the first 82 patients are presented. In addition, the baseline characteristics and short-term outcomes of the supervised cases (30 in total) were compared with the following unsupervised cases (52 cases). Moreover, we analyse the GAS results of the supervised cases by comparing the GAS form scores of the first five cases (cases 1–5) with those of the latter five cases (cases 6–10). Finally, we assess the trainee’s learning curves by presenting the GAS CUSUM charts of each surgeon for each component assessed.

For the patients recruited in this study, the preoperative assessment was the same as that described in our previous case series for the da Vinci Si system [7]. Patients with a confirmed diagnosis of rectal cancer underwent preoperative staging by computed tomography (CT) and magnetic resonance imaging (MRI). Multidisciplinary team (MDT) discussions of the clinical, radiological and pathological findings were conducted on a weekly basis prior to the start of any treatment. Preoperative chemoradiotherapy was reserved for T4 rectal tumours or those where the circumferential resection margin (CRM) appeared threatened on MRI (< 2 mm). Tumours considered resectable by total mesenteric excision (TME) with a good likelihood of clear margins did not receive any neoadjuvant treatment.

The robotic approach was the preferred approach for all patients suitable for minimally invasive rectal surgery. There were no exclusion criteria for robotic surgery that did not apply for laparoscopic surgery, and applied surgical modality (robotic or laparoscopic) was based on equipment and theatre availability. All included patients signed an informed consent allowing their data to be used for analysis and research, and the requirements for anonymisation of personal dataset by the Data Protection Act 1998 were satisfied. According to the Health Research Authority (HRA), this study was not classified to need their approval as it is an audit.

### Surgical technique

For all rectal resections, we applied a standardised technique developed through a modular approach similar to the one described for the previous generation robot (da Vinci Si®) [7]. This principle was initially developed for laparoscopic colorectal surgery [21]. Applying the modular approach enables validity through reproducibility and enhances training and research. A more detailed account, including an intraoperative video, of the surgical technique applied for robotic rectal surgery with the da Vinci Xi and ITM is given in a previous publication [20].

#### Port placement

**Fig. 1:**
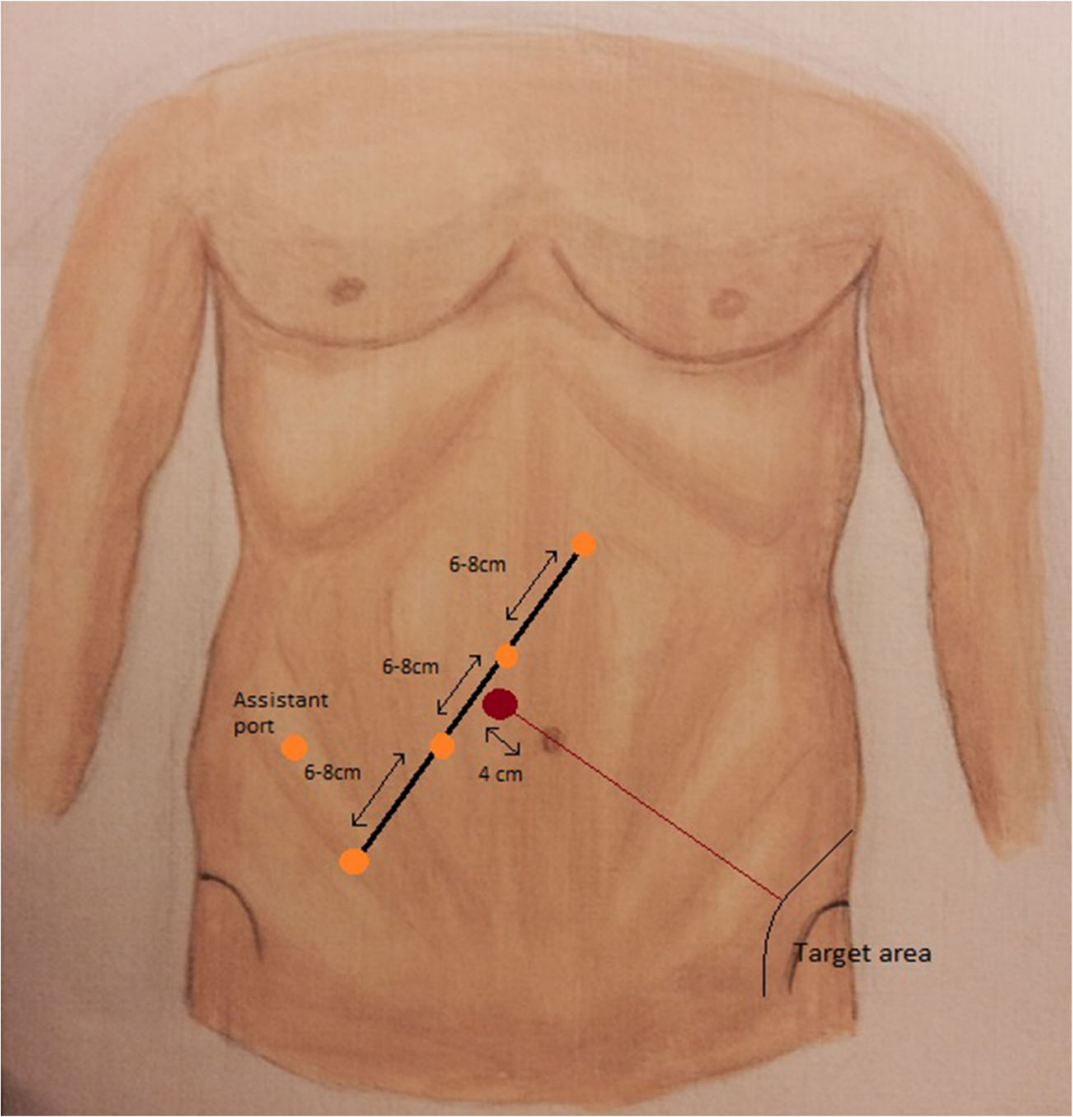
Port positions

Robotic port configuration is demonstrated in Fig. 1. Ports R1 to R4 are all placed 6–8 cm apart from each other in a straight line on the right side of the abdomen oblique to the midline. A mark is placed 4 cm to the right of the umbilicus on a line crossing the umbilicus and the camera target area (the rectosigmoid), the straight line on which the ports are placed should not cross this mark. A 12-mm robotic port is used for R4 or R3, with the remaining robotic ports being 8 mm in calibre. R4 is the most inferolateral port, and it is placed roughly two fingers superior and medial to the right anterior superior iliac spine, in a similar position recommended for laparoscopic surgery. An assistant 10-mm laparoscopic port is placed between and behind ports R3 and R4 for suction/irrigation, vessel ligation and retraction.

#### Robot docking

**Fig. 2:**
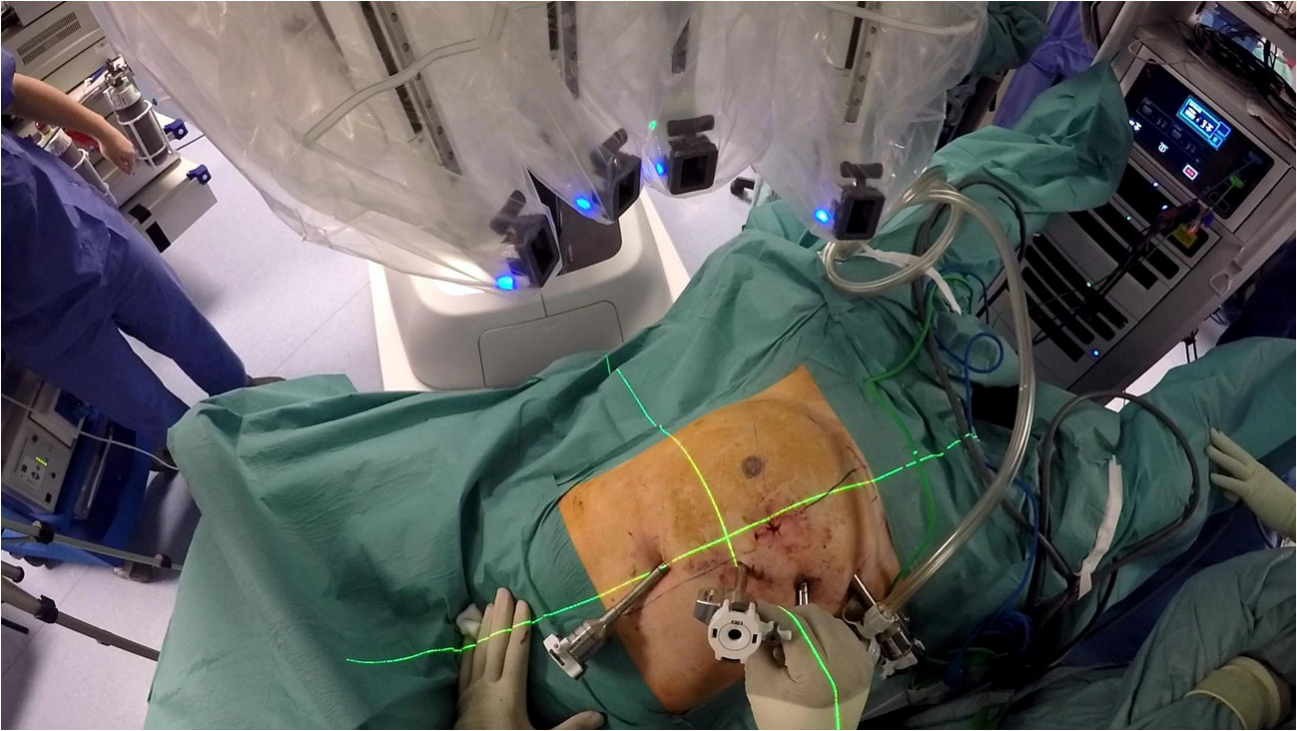
Laser target system from overhead boom

**Fig. 3:**
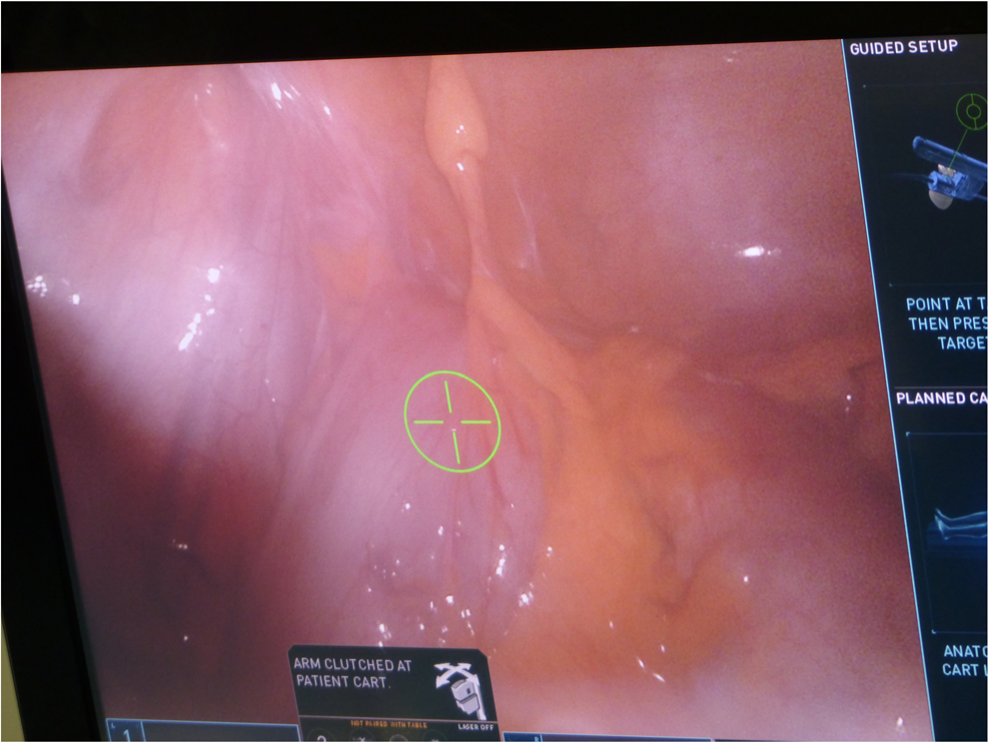
Selection of target anatomy

The patient is placed in a Trendelenburg position of approximately 22° and right tilt of 7°–9° to allow for an optimal exposure of the operative field. Following port placement, the small bowel and omentum are displaced from the operative field towards the stomach. The robotic patient cart is set to the left lower abdomen anatomy setting and brought towards the patient from the patient’s left side. The patient cart’s overhead boom projects a green target laser from the centre of its boom (see Fig. 2). The green target laser is aligned to R2. The camera is then inserted in R2, pointed towards the rectosigmoid junction area which is selected as the target anatomy (Fig. 3). The cart then automatically positions its boom in an optimised configuration. The remaining robotic arms are docked and the rest of the instruments inserted; fenestrated bipolar forceps are inserted in R1, scissors with monopolar diathermy in R3 and Cadiere forceps in R4.

#### Left colonic and splenic flexure mobilisation

Procedures commence with medial to lateral dissection followed by vascular control by ligating the main vessels. A three-step approach is used for splenic flexure mobilisation [22]. This includes (1) medial to lateral dissection towards and above the pancreas, (2) lateral colonic mobilisation and (3) separation of the omentum from transverse colon.

#### Total mesenteric excision

TME is performed in the same stepwise manner described for laparoscopic surgery and the da Vinci Si system [7, 23]. Dissection commences posteriorly and proceeds to the lateral and anterior planes in a stepwise manner, ensuring care is taken to avoid injuring the pelvic autonomic nerves.

An EndoWrist Stapler 45mm® is used to divide the rectum. Following this, the robot is undocked and the specimen is extracted through a 4–5-cm midline incision using a wound protector. A circular stapler (CDH29mm™) is used to perform the anastomosis before a flexible endoscope is routinely used to check the anastomosis and a leak test performed. Finally, a drain is inserted into the pelvis, and a defunctioning loop ileostomy is routinely performed for all patients with mid- or low-rectal tumours.

### Training programme

Before the commencement of the training programme, each surgeon was required to complete the online modules for the robotic Xi system. This included an online assessment and a 2-h course on the robotic Xi system. This was followed by simulation training, for which the surgeon was required to achieve simulation competence scores for camera targeting and suturing exercises. Following successful completion of both the system and simulation training, each surgeon enrolled in the structured programme for teaching and training run by the European Academy of Robotic Colorectal Surgery (EARCS) (see https://earcs.pt). The constituents for the training programme include (a) formal case observations during which the surgeon visits a centre of excellence to observe at least two robotic rectal resections, (b) a trainer led 2-day course which involves both animal and cadaveric models and (c) formal clinical training. The formal clinical training comprises a closely supervised programme during which the trainee performs ten robotic rectal resections. This ensures a similar baseline training with equitable access to procedural skill acquisition.

Each operation is divided into modules similar to those previously described for laparoscopic rectal surgery [21]. This makes both training and de-briefing simpler. Additionally, video recordings from each training cases were used to give direct feedback to the trainees. Each operation was scored in real time by the trainer using a GAS form. Once the trainees reached a level of competence as assessed by the blind video assessment of the EARCS, the surgeons embark upon solo performance of their cases.

#### Global assessment score form

**Fig. 4:**
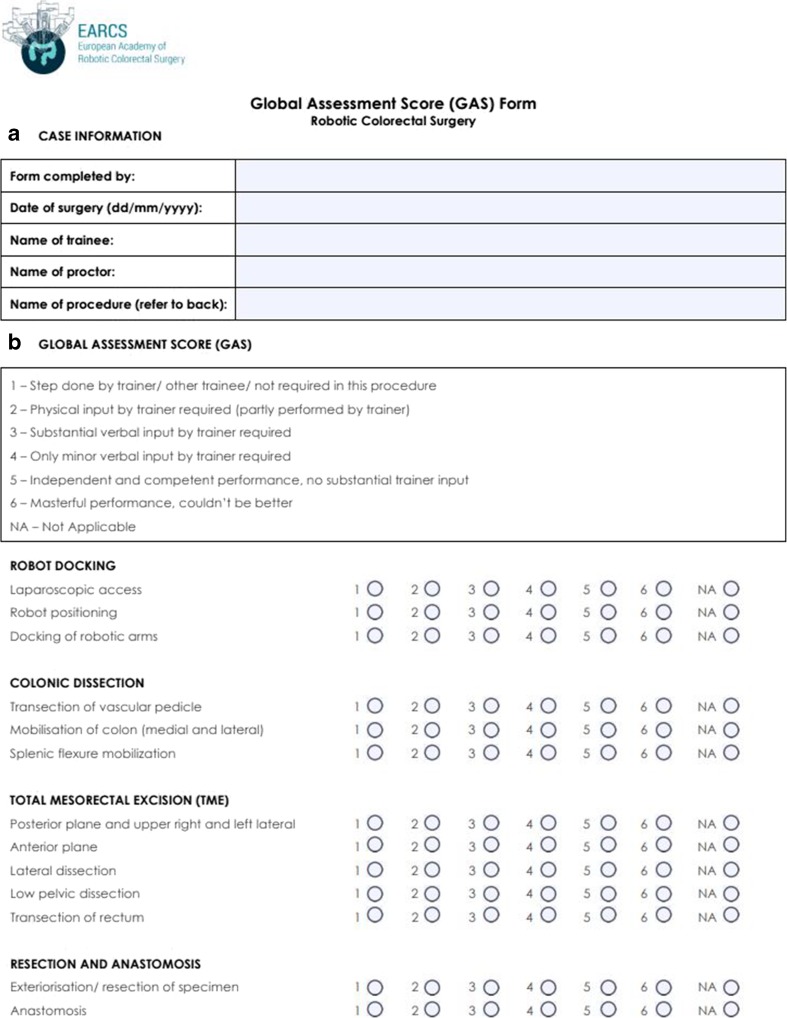
GAS form

The GAS form used by EARCS contains four modules, each containing several components. These include (1) robotic docking, (2) colonic dissection, (3) TME and (4) resection and anastomosis. In Fig. 4, we present the GAS form used by the EARCS faculty, including the components of each module. Each component is scored from 1 to 6 (or not applicable if the step is not performed) with the scores given corresponding to the competence levels presented in the GAS form in Fig. 4. As demonstrated, the higher the score, the higher the competence level for each component.

### Statistical analysis

Data was analysed using IBM SPSS version 24 (SPSS Inc., Chicago, IL, USA) and Microsoft Excel 2010™. Non-parametric data was expressed as median with interquartile range (IQR) and parametric data as mean with standard deviation (SD). Presented data is rounded up to whole numbers. Baseline demographic and clinical characteristics were compared using *χ*^2^ test or Fisher’s exact test for categorical variables, Mann-Whitney *U* test for non-parametric continuous variables and *t* test for parametric continuous variables. *p* values of < 0.05 were considered statistically significant.

For purposes of statistical analysis, not applicable GAS form entries were assigned a value equal to that of the previous GAS form in the corresponding component. There were only 10 (out of 390) not applicable entries.

#### Cumulative sum charting

GAS CUSUM curves for each component were charted in order to assess the learning curves of the trainee surgeons [24, 25]. This allowed us to investigate whether ten cases were sufficient for each surgeon to reach competence in each component.

For the construction of the GAS CUSUM charts, we used a target score of 5, which is equitable to independent and competent performance. Therefore, the CUSUM score is the cumulative sum of the GAS minus 5. In the CUSUM chart, the *x*-axis represents the consecutive cases and the *y*-axis the CUSUM score. The CUSUM curves descend when the set target is not reached, which reflects an ongoing learning process. When the curve plateaus or ascends, the target is achieved or superseded, representing the end of the learning process.

## Results

Eighty-two consecutive patients who underwent robotic rectal surgery with the da Vinci Xi® system between November 2015 and September 2017 were recruited to this study. Fifty-nine patients were operated in the UK centre and 23 at the centre in Portugal.

### Baseline characteristics

Their baseline characteristics of all patients operated with the da Vinci Xi and ITM are shown in Table 1. Fifty-one patients were male and thirty-one female. Median age was 69 (IQR 59–76) and median BMI 29 (IQR 24–30). Twenty-two patients (27%) received neoadjuvant chemoradiotherapy.

**Table 1:** Baseline characteristics

	Total (*n* = 82)
Sex	
• Male	51 (62%)
• Female	31 (38%)
Median age (IQR)	69 (59–76)
Median BMI (IQR)	28 (24–30)
ASA grade	
• I	6 (7%)
• II	60 (74%)
• III	15 (19%)
Neo adjuvant chemoradiotherapy	22 (27%)
T stage (MRI)	
• T1	9 (11%)
• T2	18 (23%)
• T3	45 (56%)
• T4	8 (10%)

### Perioperative and postoperative outcomes

The peri- and postoperative characteristics of the patients who underwent robotic rectal surgery are shown in Table 2. Seventy-two patients (88%) had anterior resections and ten (12%) had abdominoperineal excisions. There were no conversions to open, no anastomotic leaks and no 30-day mortality. Mean operative time was 288 min (SD 63), median estimated blood loss is 20 ml (IQR 20–20) and length of stay is 5 days (IQR 4–8). The median time the patients spent in the reverse Trendelenburg position during splenic flexure mobilisation was 30 min (IQR 21–35). Eighty-one (99%) circumferential resection margins were R0.

**Table 2:** Perioperative and postoperative outcomes

	Total (n = 82)
Procedure	
• Anterior resection	72 (88%)
• Abdominoperineal excision	10 (12%)
Conversion to open	0
Mean operative time (SD)	288 (63) (*n* = 59)
Median reverse Trendelenburg time in minutes (IQR)^1^	30 (21–35)
Median blood loss in ml (IQR)	20 (20–20)
Median length of stay in days (IQR)	5 (IQR 4–8)
30-day readmission	3 (4%)
30-day reoperation	5 (6%)
Anastomotic leak	0
30-day mortality	0
Lymph node yield (IQR)	21 (16–30)
R0	81 (99%)

^1^First 20 patients only

There were three 30-day readmissions (4%) and five 30-day reoperations (6%). Readmission indications included a patient with a wound infection, a patient with a high output stoma and a patient who presented 4 weeks after her operation with signs of small bowel obstruction and a patient who subsequently did not require any surgery and settled with conservative management.

Reoperation indications included a patient with small bowel obstruction that required a laparoscopic adhesiolysis, a patient with small bowel obstruction at the ileostomy site whose ileostomy was reversed, a patient with postoperative intra-abdominal bleeding that was taken to theatre for laparoscopy 2 days postoperatively, a patient with an abscess that had a radiological drain inserted and a patient with a chyle leak which was discharging chyle from a pelvic drain. Initially, the drains’ contents were diagnosed as being Fortisip® (nutrition drink) since the patient was taking oral fluids, and therefore, a peptic perforation was suspected. The patient underwent a diagnostic laparoscopy, and no viscus perforation was found, confirming a chyle leak.

### First ten cases of each surgeon (supervised) vs subsequent cases (solo)

Since there were three surgeons participating in this study, there were a total of 30 cases performed under the supervision of a single trainer (surgeon ACP) using the standardised approach previously described [7, 20]. Following the completion of supervised training, a further 52 cases were performed independently by all three surgeons.

The baseline characteristics and peri- and postoperative outcomes of these patients are presented in Tables 3 and 4. There were no differences in the baseline characteristics or short-term surgical outcomes between the two groups with the exception of operative time. Mean operative time improved in the latter cases by 36 min (*p* = 0.038).

**Table 3:** Baseline characteristics of supervised vs independent cases

	First 10 cases for each surgeon/supervised (*n* = 30)	Independent cases (*n* = 52)	*p* value
Sex			
• Male	17 (57%)	34 (65%)	.433^c^
• Female	13 (43%)	18 (35%)	
Median age (IQR)	70 (59–78)	69 (59–75)	.665^m^
Median BMI (IQR)	27 (24–29)	28 (25–30)	.558^m^
ASA grade			
• I	3 (10%)	3 (6%)	.418^c^
• II	19 (66%)	41 (79%)	
• III	7 (24%)	8 (15%)	
Neo adjuvant chemoradiotherapy	6 (20%)	16 (31%)	.289^c^
T stage (MRI)			
• T1	6 (20%)	3 (6%)	.126^c^
• T2	4 (13%)	14 (28%)	
• T3	18 (60%)	27 (54%)	
• T4	2 (7%)	6 (12%)	

c, chi square; m, Mann-Whitney *U*

**Table 4:** Perioperative and postoperative outcomes of supervised vs independent cases

	First 10 cases for each surgeon/supervised (*n* = 30)	Independent cases (*n* = 52)	*p* value
Procedure			
• Anterior resection	27 (90%)	45 (87%)	.645^c^
• Abdominoperineal excision	3 (10%)	7 (14%)	
Conversion to open	0	0	
Mean operative time in minutes (SD)	311 (55) (*n* = 20)	275 (65) (*n* = 39)	*.038* ^t^
Median blood loss in ml (IQR)	20 (20–20)	20 (20–20)	.726^m^
Median length of stay in days (IQR)	5 (4–8)	5 (4–8)	.942^m^
30-day readmission	1 (3%)	2 (4%)	1.000^f^
30-day reoperation	2 (7%)	3 (6%)	1.000^f^
Anastomotic leak	0	0	
30-day mortality	0	0	
Lymph node yield (IQR)	22 (17–31)	20 (15–30)	.508^m^
R0	30 (100%)	51 (98%)	1.000^f^

c, chi square; t, *t* test; m, Mann-Whitney *U*; f, Fisher’s exact test

Italic entry is statistically significant

### GAS form scores and GAS CUSUM charts

The GAS form scores of the first five cases (cases 1–5) and latter five cases (6–10) of each surgeon are presented in Table 5. There was a significant improvement (*p* = 0.000) in median scores in the latter five cases in all the components assessed in the GAS form. Moreover, the median scores of cases 6–10 demonstrate that ten supervised cases were sufficient for the surgeons to perform robotic rectal surgery independently.

**Table 5:** GAS form analysis of first and latter five cases (cases 1–5 vs 6–10) performed by the three participating surgeons

	Median GAS form scores from first five cases of each trainee (*n* = 15)	Median GAS form scores from latter five cases of each trainee (*n* = 15)	*p* value (Mann-Whitney *U*)
Robot docking			
Laparoscopic access	3 (3–4)	5 (5–6)	.000
Robot positioning	3 (2–4)	5 (5–6)	.000
Docking of robotic arms	3 (2–4)	5 (5–6)	.000
Colonic dissection			
Transection of vascular pedicle	3 (2–3)	5 (4–5)	.000
Mobilisation of colon	3 (2–3)	5 (4–5)	.000
Splenic flexure mobilisation	2 (2–3)	5 (4–5)	.000
TME			
Posterior plane and upper right and left lateral plane	3 (2–3)	5 (5–5)	.000
Anterior plane	2 (2–3)	5 (4–5)	.000
Lateral dissection	2 (1–3)	5 (4–5)	.000
Low pelvic dissection	2 (2–2)	4 (4–5)	.000
Transection of rectum	3 (2–4)	5 (5–6)	.000
Resection and anastomosis			
Exteriorisation/resection of specimen	4 (3–5)	6 (5–6)	.000
Anastomosis	4 (3–5)	5 (5–6)	.000

**Fig. 5:**
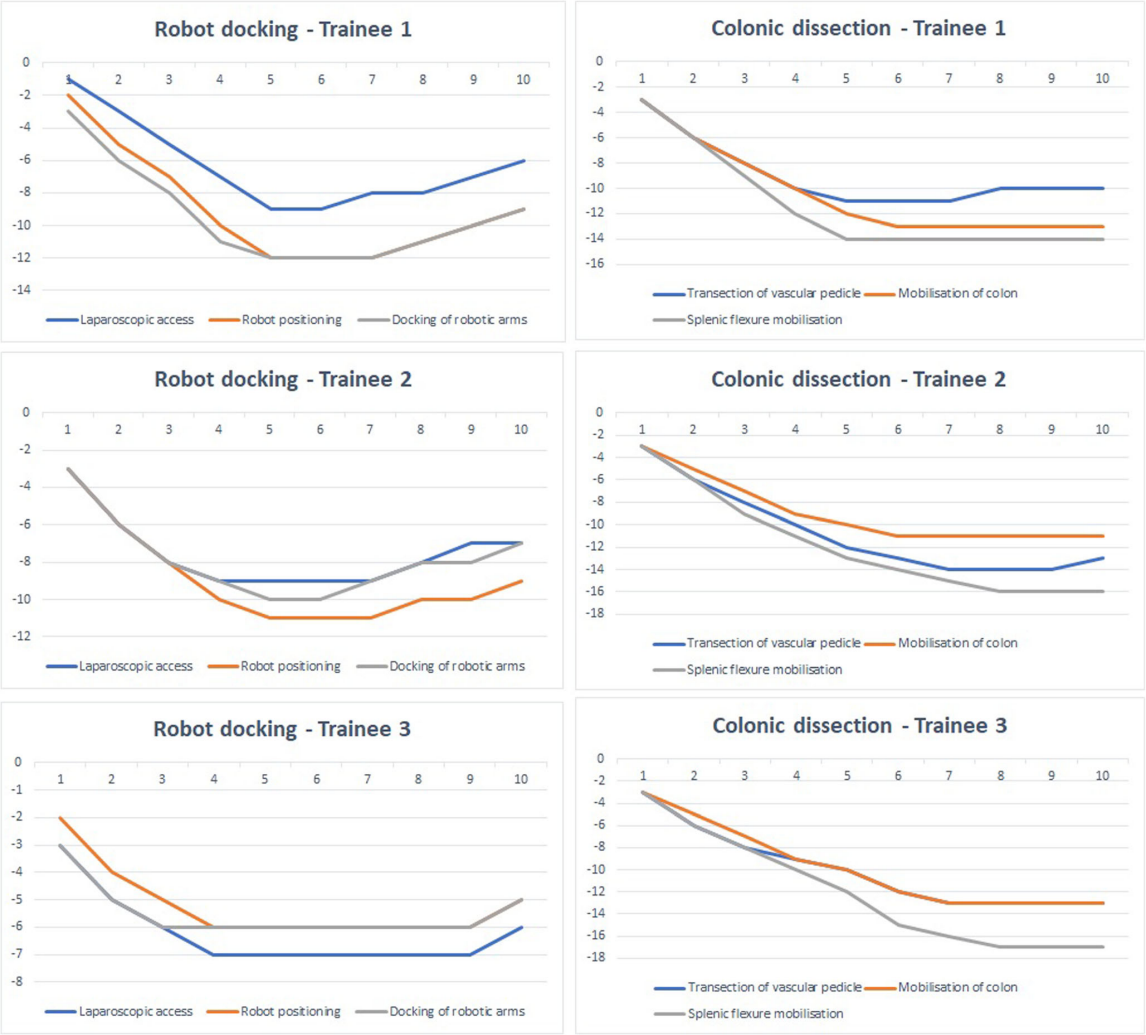
GAS CUSUM charts for modules 1 and 2

**Fig. 6:**
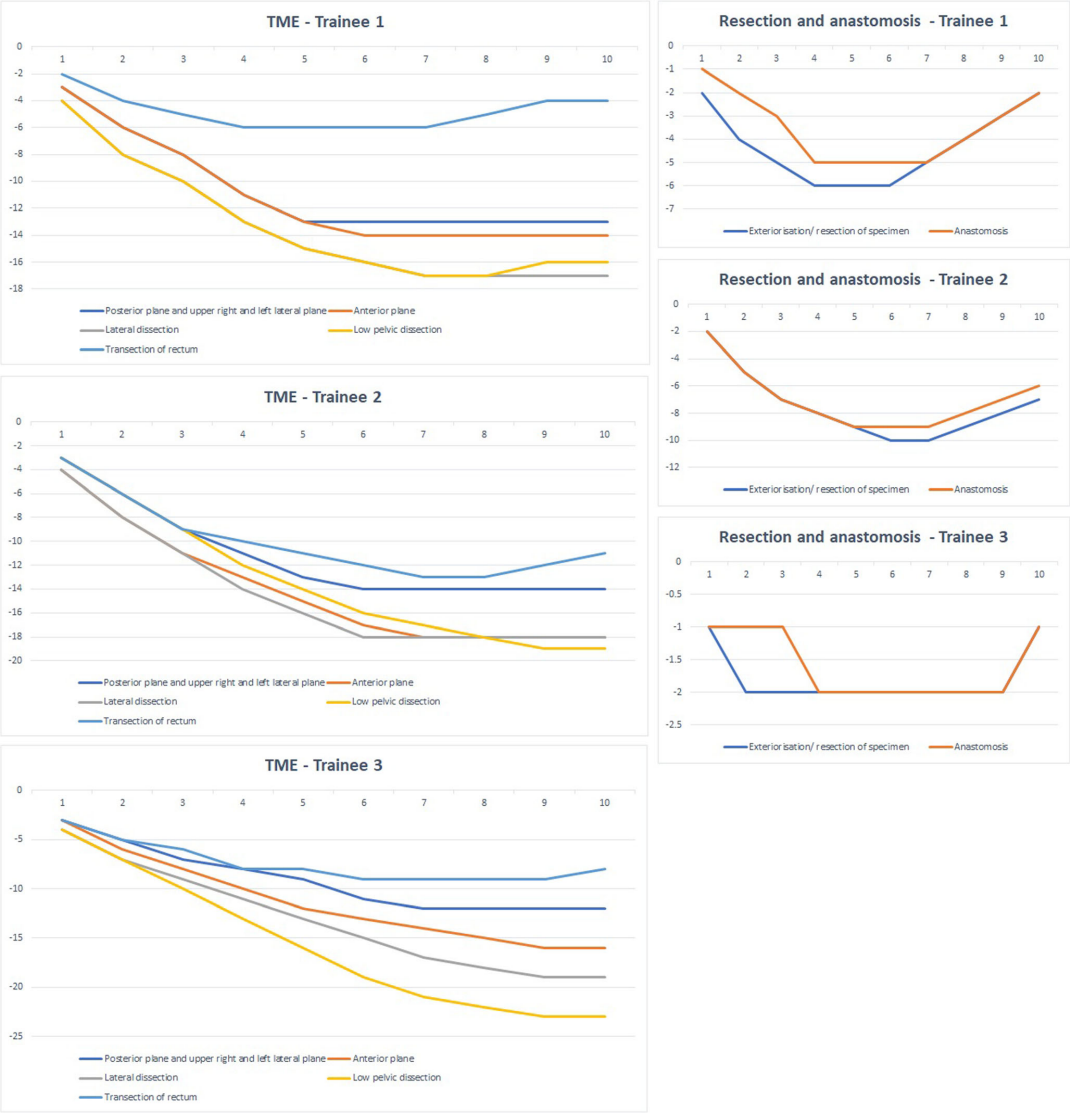
GAS CUSUM charts for modules 3 and 4

The GAS CUSUM charts of the robot docking and colonic dissection modules (1 and 2) are presented in Fig. 5 and those of the TME and resection and anastomosis modules (3 and 4) in Fig. 6. Ten cases were enough to reach competence in all the components assessed in the GAS forms, as demonstrated by the plateauing or ascending of the CUSUM curves in all the CUSUM charts. This was the case for all three surgeons participating in this study.

## Discussion

Minimally invasive surgery has become the gold standard of care for elective colonic surgery. However, its role for rectal resection is still a matter of great interest and debate. This is reflected in at least two recently published randomised controlled trials (ACOSOG [26] and ALaCaRT [27]) where the oncological equivalence of laparoscopic to open rectal surgery is debated. This has led colorectal surgeons to seek alternative options of minimally invasive surgery for patients with rectal cancer. Robotic rectal surgery is increasingly gaining acceptance amongst colorectal surgeons albeit in its early stages with a variety of techniques described in the literature [5–12, 28].

In order to achieve acceptable clinical outcomes using any new technology, there are two components for success: firstly, competence in using the instruments and equipment available; secondly, a structured training programme which ensures patient safety and good clinical outcomes. Modular approaches to surgery were first described and effectively used to teach endoscopic prostatectomy and coronary artery bypass grafting [29, 30]. This method was successfully applied at the National Training Programme for Laparoscopic Colorectal Surgery (LAPCO) in England [31]. Furthermore, GAS tools, which were successfully applied in LAPCO, increase assessment objectivity and help give constructive feedback. LAPCO was used successfully as a model for an assessment-based training programme, which was successfully applied in this study.

The success of training colorectal surgeons in England within the national training programme (LAPCO) is evident from the increasing uptake of laparoscopic colorectal surgery from 10% to over 50% since its implementation [32]. The long learning curve often associated with learning new technologies and techniques can be shortened using the concept of standardisation and the structured training programme. Currently, structured training in robotic rectal surgery with a blind assessment process in the end is being used by EARCS to train surgeons in Europe. The model includes initial system training, case observations, a 2-day animal model and cadaveric course and supervised training for up to ten cases by the faculty. The progress during the training is mapped by using GAS forms. Clinical and pathological outcome data is collected. Following the completion of the supervised training, two unedited videos performed by the trainees are blindly assessed by the faculty to judge competence. To date, blinded video assessment has been shown to be the most consistent and unbiased way to assess a surgeon’s competence [31].

In this study, we have demonstrated that good clinical outcomes for robotic rectal surgery can be achieved by such a structured, equitable and available training programme without compromising patient safety. In addition, we have demonstrated that short-term surgical outcomes remain similar when the trained surgeons start performing independent cases following the initial ten supervised cases. Finally, by analysing the GAS forms and the GAS CUSUM charts, we have demonstrated that ten proctoring cases are probably sufficient for the trainee surgeons to reach a competent level for performing robotic single-docking rectal surgery with the Xi and ITM.

As far as we are aware, this is the first study reporting the short-term surgical outcomes of colorectal surgeons taking part in a structured robotic rectal surgery training programme. Although the feasibility of robotic rectal surgery with the da Vinci Xi has been confirmed in previously published studies [33–36], only one of which includes the use of the ITM [36], the sample sizes of these studies are relatively small. This study presented here is the largest case series of robotic rectal surgery with the da Vinci Xi and is unique in describing the implementation of a structured training programme for this approach.

The training pathway for each trainee was mapped by the use of GAS forms which were filled in real time at the end of each case by the trainer. This tool provided a robust timeline and progress map of the trainees during their training pathways. Analysis of the GAS forms of the three surgeons participating in this study demonstrated clear progress in the scores from the first to the latter five cases and revealed that by the latter five cases, the surgeons achieved median scores reflecting independent practice (see Table 5). Furthermore, the GAS CUSUM charts (see Figs. 5 and 6) exhibited a plateau by ten cases for all the individual components of robotic rectal surgery for all three surgeons. These findings suggest that as long as the formal training pathway for robotic rectal surgery is undertaken, ten proctoring cases are probably sufficient for trainee surgeons to learn how to independently perform robotic rectal surgery with the da Vinci Xi. It is worth mentioning that the trainee surgeons in this study had considerable experience in laparoscopic rectal resections. One could argue that this eased their performance and shortened their learning curves in robotic surgery, although whether laparoscopic skills are transferable to robotic surgery is a subject of debate, with studies presenting conflicting results [37, 38]. It would be interesting to see whether surgeons transitioning from open to robotic surgery, with limited prior laparoscopic surgery, would have similar learning curves in a robotic rectal surgery training programme.

Examining the short-term surgical outcomes of all 82 cases, our results infer that by standardising the technique and applying a modular approach, the single-docking procedure with the da Vinci Xi and ITM is reproducible, safe and feasible. However, the operative time in our series could be considered relatively high, and in fact, it was higher than that in the da Vinci Si series described by Ahmed et al. [7], a study describing 100 consecutive cases performed by two surgeons, one of which is the trainer for this study. This may be because the three surgeons in our study, as well as the theatre stuff, are still in the early phase of their respective learning curves. In the literature, operative time with the da Vinci Xi ranges from 237 to 289 min [33–36], which is similar to the operative time reported in our case series. Considering these are all early studies including small sample sizes, it is likely that operative time will decrease as surgeons and theatre staff further progress in their learning curves with the robotic systems. This may be why the operative time in our study was shorter in the subsequent, independent cases. It is worth noting that further studies where surgeons switched from the Si to the Xi system report an improvement in their operative time [15, 39].

The main strengths of this study are its larger sample size, its novelty in terms of describing the training pathway for robotic rectal surgery and the fact that the data was collected from two centres in two countries. We need to acknowledge the limitations of this study, which mainly lie in the retrospective nature of this study and the lack of reporting of any functional data. Furthermore, although the number of supervised cases performed is equal between the participating surgeons (ten each), there is a variability in the number of independent cases performed by each surgeon after completion of their training. This could add an element of observation bias when comparing the supervised with the unsupervised cases.

In conclusion, all three trainee surgeons in our study were put through an assessment-based structured training programme. It allowed for consistently good outcomes with all three trainees having as a common denominator a single trainer using the same standardised training technique. This programme is currently run by EARCS across Europe. As the data for a greater number of surgeons becomes available, it would be interesting to analyse and see if similar conclusions could be reached for a larger group of trainees. We eagerly await the results of the European wide programme in the near future.
